# Potential Universal Engineering Component: Tetracycline Response Nanoswitch Based on Triple Helix-Graphene Oxide

**DOI:** 10.3390/mi13122119

**Published:** 2022-11-30

**Authors:** Luhui Wang, Yue Wang, Mengyang Hu, Sunfan Xi, Rong Liu, Meng Cheng, Yafei Dong

**Affiliations:** 1 College of Life Science, Shaanxi Normal University, Xi’an 710119, China; 2 College of Computer Sciences, Shaanxi Normal University, Xi’an 710119, China

**Keywords:** tetracycline, triplex structure, graphene oxide, specificity detection, fluorescent nanoswitch

## Abstract

The overuse of antibiotics can lead to the emergence of drug resistance, preventing many common diseases from being effectively treated. Therefore, based on the special composite platform of P1/graphene oxide (GO) and DNA triple helix, a programmable DNA nanoswitch for the quantitative detection of tetracycline (TC) was designed. The introduction of GO as a quenching agent can effectively reduce the background fluorescence; stabilizing the trigger strand with a triplex structure minimizes errors. It is worth mentioning that the designed model has been verified and analyzed by both computer simulation and biological experiments. NUPACK predicts the combined mode and yield of each strand, while visual DSD flexibly predicts the changes in components over time during the reaction. The feasibility analysis preliminarily confirmed the realizability of the designed model, and the optimal reaction conditions were obtained through optimization, which laid the foundation for the subsequent quantitative detection of TC, while the selective experiments in different systems fully demonstrated that the model had excellent specificity.

## 1. Introduction

Antibiotics are one of the most important medical discoveries in the 20th century, and they have made a great contribution to the treatment of bacterial infections and the animal breeding industry [1]. On the other hand, the misuse and accumulation of antibiotics poses a threat to human health and the ecosystem. Tetracycline (TC) is a broad-spectrum antibiotic produced by Streptomyces, which is often used as a pharmaceutical additive and is widely used in human treatment, animal breeding, and veterinary medicine due to its advantages such as effective antibacterial performance, low price, and reduced side effects [2,3]. Excessive TC from different sources introduced into the environment, especially in water resources, can cause serious environmental problems [4,5]. In addition, TC may accumulate in the human body through the food chain, leading to adverse consequences such as bacterial drug resistance and tetracycline teeth [6,7]. Therefore, although the efficacy of TC in medical care cannot be ignored, its harm to the environment and health cannot be underestimated.

Traditional detection methods of TC include liquid chromatography (HPLC) [8], capillary electrophoresis (CE) [9], liquid chromatography tandem mass spectrometry (LC-MS/MS) [10], immunoassay [11], and enzyme-linked immunosorbent assay (ELISA) [12]. These methods offer high accuracy, sensitivity, and reliability in standard analysis in well-equipped laboratories with the analysis being limited by expensive instruments, cumbersome sample extraction procedures, and a lack of experienced operators. Therefore, in recent years, biosensors for the detection of different substances have developed rapidly due to their advantages of convenience, cheapness, and rapidity. Nanomaterials often have special properties, and the combination with traditional biosensors can realize different degrees of transformation and improve the diversity of biosensors. Specifically, GO and its derivatives can effectively adsorb single strand DNA (ssDNA) and quench the fluorescence it labels or carries by fluorescence resonance energy transfer (FRET), which allows GO to effectively reduce background noise when used in novel fluorescence sensors [13,14]; gold nanoparticles can aggregate and develop color, and different color changes can occur in different degrees of aggregation, thus they have a place in the new colorimetric sensors [15,16]; a gold electrode plate can firmly combine with a sulfhydryl-modified DNA strand to generate electrical changes through different structural DNA and are an indispensable part of the electrochemical biosensors [17,18]. Based on these studies, it is necessary to design a new nanoswitch with a simple operation that combines nanomaterials and biological properties for rapid and sensitive detection of TC.

DNA aptamers are short fragments of DNA repeatedly screened from a library of random oligonucleotide sequences that are obtained from systematic evolution of ligands by exponential enrichment (SELEX) technology [19,20,21]. Aptamers have a series of advantages, such as easy artificial synthesis, large-scale chemical production, high sensitivity, high selectivity, reversible thermal denaturation, easy labeling, fixation, signal transduction, and regeneration [22,23]. Because aptamers have high specificity for a variety of targets such as small molecules, proteins, metal ions, or cell surfaces [24,25,26], they have been used to design a variety of sensing devices, including electrochemical sensors, fluorescent sensors, and colorimetric sensors [27,28]. In this paper, we use an 8-mer ssDNA aptamer fragment truncated by Kwon et al. in 2014, which has been shown to have high affinity for four different TCs (tetracycline, oxytetracycline, doxycycline, and chlortetracycline) [29].

**Figure 1:**
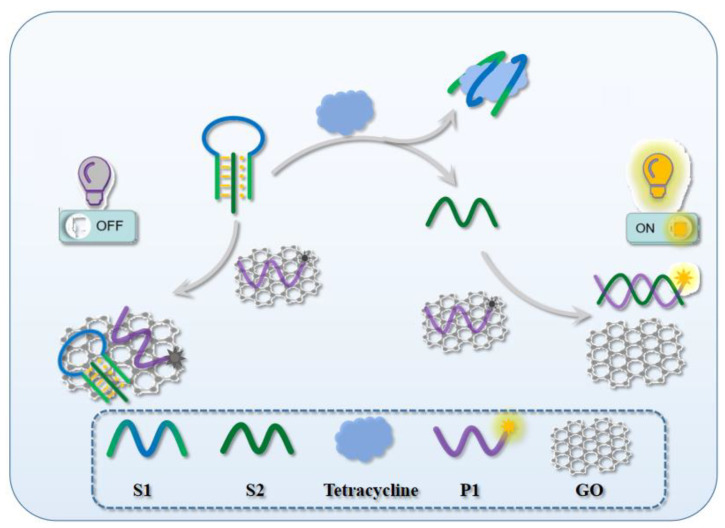
Principle of TC-responsive nanoswitch.

Based on the above research status, we designed an engineering biosensor based on triple helix structure and GO to achieve the purpose of rapid detection of TC through the fluorescence change (OFF/ON) in the system. Unlike the common DNA double helix, triple helix nucleic acid is formed by Watson–Crick base pairing and a Hoogsteen hydrogen bond interaction, which means that the insertion strand is fixed by the two effects and is more stable, so as to avoid the false-positive as much as possible. Specifically, there are three types of ssDNA in the system, named S1, S2, and P1 (Figure 1). The double ends of S1 have the same base sequence, with the middle part being the specific binding region of TC. S2 is complementary with S1 end sequences, and under acidic conditions they can be wound to form a triple helix structure. The probe P1 labeled with fluorescence FAM is a complementary sequence of S2, since S2 is fully bound by S1 in the system, P1 is in a single- stranded state due to the absence of complementary strand binding, which is easily adsorbed by GO and quenching the labeled fluorescence, so the fluorescence intensity in the whole system is weak (FLUORESCENCE OFF). When detecting the presence of target TC, it will first combine with the loop part of the S1–S2 triple helix hairpin and open the triple helix structure. At this time, S2 will be easily attracted by P1 to form a P1-S2 double-strand structure that helps P1 leave to the GO surface and the fluorescence value of the system will be restored (FLUORESCENCE ON). Depending on the variation in the fluorescence intensity of the system, the designed model can specifically detect TC. 

## 2. Materials and Methods

### 2.1. Reagents and Materials

All of the DNA (sequence in Table S1) and DNA makers were purchased from Sangon Biotechnology Co., Ltd. (Shanghai, China) and purified by PAGE and ULTRAPAGE, and all of them were dissolved in ultrapure water as stock solutions (10 μM). TC, amoxicillin, and ofloxacin were purchased from Beijing Shiji Aoke Biotechnology Co., Ltd. The GO dispersion (XF020) was purchased from Nanjing XFNANO Materials Technology Co., Ltd. (Nanjing, China). SYBR Green I and Stain-All dye were purchased from Beijing Solarbio Science & Technology Co., Ltd, (Beijing, China)

### 2.2. Construction of Triple Helix DNA and P1/GO Platform 

S1 and S2 were mixed in 10 mM Tris-HCl buffer (pH 6.2, 0.1 M NaCl, 5 mM MgCl2. 5 mM KCl, and 0.5 mM EDTA), and in order to reduce the leakage of the trigger strand, the S1 concentration was greater than S2 (concentration ratio 2:1, the S1–S2 triple helix hairpin final concentration was 200 nM). The mix solution was incubated at 95 °C for 5 min and then slowly cooled to room temperature to form the stable S1–S2 triple helix structure. 

P1 (100 nM) and GO dispersion (25 μg/mL) were mixed in Tris-HCl buffer and reacted at room temperature for 1 h to obtain the P1/GO system with sufficient quenching of P1-labeled FAM fluorescence for subsequent experiments.

### 2.3. Fluorescence Signal Detection 

At a temperature of 25 °C, mix TC (200 nM) with S1–S2 solution and incubate the mixture for 1 h to release S2. Then, add GO/P1 dropwise to the reaction mixture described above. After approximately 30 min of incubation at room temperature the final solution was used for fluorescence detection. For FAM-labeled substrates, use the Multifunctional Microplate Detector (EnSpire ELISA; PerkinElmer, Waltham, MA, USA) scan at 492 nm excitation and 518 nm emission. In the SYBR Green strategy, the fluorescence results were obtained from the multifunctional microplate reader (SLXFA; BioTek, Winooski, VT, USA) using green filters (excitation 485 ± 20, emission 528 ± 20).

### 2.4. Polyacrylamide Gel Electrophoresis (PAGE) 

A 15% polyacrylamide gel was prepared by mixing 7.875 mL distilled water, 5.625 mL of 40% gel solution, 1.5 mL 10× TAE buffer, 112.5 μL 10% APS, and 11.25 μL TEMED. Then, the PAGE experiment was carried out in 1× TAE buffer at 120 V for 1.5 h. The gel was dyed in a Stain-All dye solution for about 20 min, and, finally, the imaging was observed using the JS-680D automatic gel imaging analyzer (Peiqing, Shanghai, China). Grayscale analysis was performed with Image J.

## 3. Results and Discussion

### 3.1. Simulation Experiment

Before the wet experiment, we used computer simulations to perform a preliminary simulation of the experimental process to estimate the suitable experimental temperature and the approximate time required for each stage of the reaction. It should be noted that in the actual experiment, the S1 concentration is increased in order to avoid S2 leakage, but the excess S1 will be adsorbed by GO and it is difficult to participate in the subsequent reaction, so in order to simulate the GO effect and make the results easier to observe, the pictures in the text are the results of the simulation experiments when the concentration of each component is the same.

**Figure 2:**
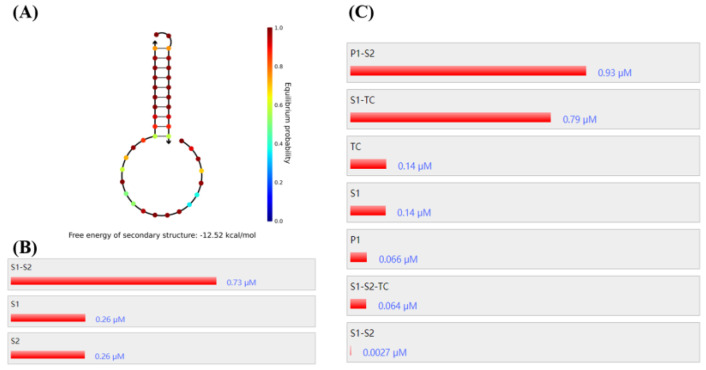
NUPACK simulates S1–S2 stability: (**A**), S1–S2 binding rate, (**B**) and component concentration in the presence of TC (**C**).

Firstly, NUPACK [30] was used to simulate the strand combination. Due to the limitation of the software, the pH value cannot be set, but it can be seen that in the neutral case, the S1–S2 stable structure can be achieved by a single side strand with a combined efficiency of more than 70% (Figure 2A,B); it can be inferred that the triple helix structure can exist more stably and efficiently under verified pH conditions. Then, the simultaneous presence of TC, S1, S2, and P1 in the system was simulated. It can be seen that two products, double strand P1-S2 labeled with fluorescent and S1-TC complex, were mainly formed (Figure 2C), which was in line with the expected results. In the supporting information, the rationality of replacing TC with TC corresponding sequence is discussed (Figure S1)

**Figure 3:**
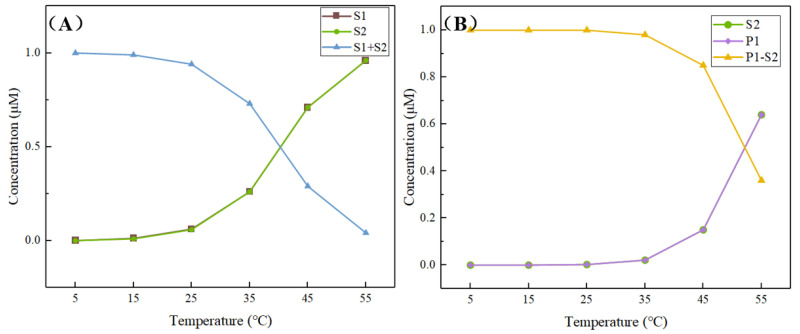
NUPACK simulates the strand binding at different temperatures. S1–S2 stability (**A**) and P1-S2 stability (**B**).

The binding of S1–S2 and P1-S2 at temperatures of 5–55 °C was further simulated to determine the optimal reaction temperature. As can be seen from Figure 3A, the S1–S2 structure has high yield and stable binding at 25 °C, but the binding capacity begins to decrease when the temperature reaches 35 °C, the binding rate is less than 50% at 45 °C, and when the temperature reaches 55 °C, the S1 and S2 strands are completely released into two independent single strands. On the other hand, the trend of P1-S2 yield with temperature is roughly similar to that of S1–S2 (Figure 3B), with the main turning point being 45 °C. In order to prevent spontaneously unwinding of S1–S2 and affecting the detection effect when the temperature is too high, 25 °C was selected as the optimal temperature for the subsequent reaction. In addition, because the above two complexes are synthesized from two substrates, the trend of substrate concentration is similar and overlapping. The results of the simulation experiment with the wet experimental concentration are displayed in the supporting information (Figure S2).

**Figure 4:**
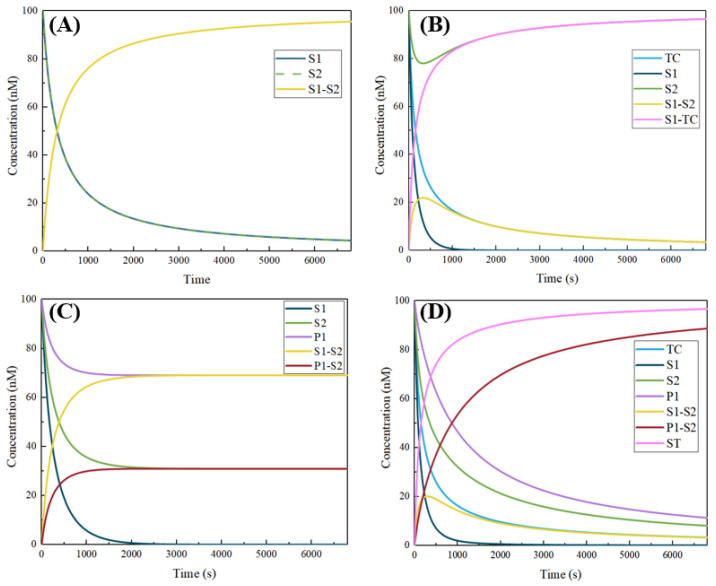
Visual DSD simulates the changes of each component concentration in the system when only S1 and S2 exist (**A**); S1, S2 and TC exist (**B**); S1, S2 and P1 exist (**C**); and all components exist (**D**).

Using Visual DSD [31] to simulate the reaction process, we can clearly see the content changes of different components in the system. Specifically, when there are only S1 and S2 in the system, their concentrations decrease synchronously to form a sufficient amount of triple helix structure S1–S2 (Figure 4A); when target TC is added in the former case, the concentration of triple helix structures increases first and then decreases, and the contents of S1-TC and released S2 in the final system are at a high level (Figure 4B). In the presence of S1, S2, and P1, most of S2 forms the triple helix structure with S1, and a small amount reacts with P1 to produce a P1-S2 double strand (Figure 4C). However, compared with the target in the system, the concentration of the formed double strand is within the acceptable limits when the target is present in the system (Figure 4D). The results of the simulation experiment with the wet experimental concentration are displayed in the supporting information (Figure S3). In addition, from the simulation process, it can be seen that the reaction of different systems can approximate to a steady state in 5400 s. 

### 3.2. Feasibility Study 

**Figure 5:**
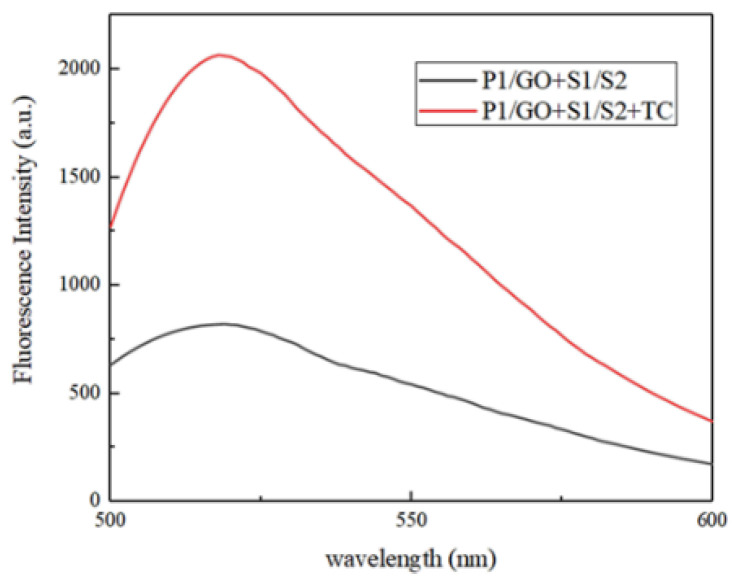
Fluorescence spectra of the feasibility study for TC detection.

The first step of experimental verification was a feasibility analysis. The fluorescence spectral result shown in Figure 5 is obtained under different conditions. In the absence of target TC, the fluorescence value was low (black curve), which is due to the stable presence of the triple helix structure while P1 is the ssDNA state, so the labeled FAM fluorescence is quenched by GO. The fluorescence increased significantly after the addition of target TC, which represents the fact that after TC binds to the aptamer S1, result in S2 strand is released and can be more fully hybridized with P1, so this affects FAM fluorescence recovery because it is far away from GO (red curve). The two curves with obvious differences can reasonably distinguish whether there is a target, which is a valid description of the scheme. 

**Figure 6:**
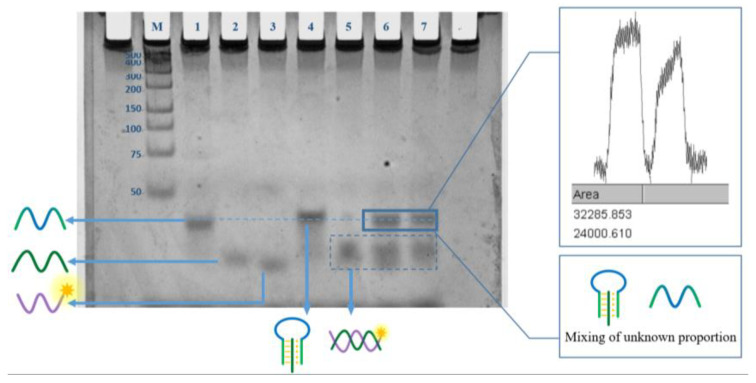
Polyacrylamide gel electrophoresis results. M: maker; lane 1: S1; lane 2: S2; lane 3: P1; lane 4: S1 + S2; lane 5: S2 + P1; lane 6: TC + S1–S2 + P1; lane 7: S1–S2 + P1.

In addition, the polyacrylamide gel electrophoresis (PAGE) experiment also proved the feasibility from another angle. As shown in the Figure 6, lanes 1, 2, and 3 are the cases where S1, S2, and P1 exist alone, respectively, as a reference for subsequent reactions. As can be seen from lane 4, when S1 and S2 are present, a slightly delayed band (S1–S2) will be formed, and the corresponding bands of S1 and S2 disappear, indicating the formation of the S1–S2 structure. Lane 5, when S2 and P1 exist at the same time, it can be seen that new P1-S2 bands are generated. In the presence of TC in lane 6, more S1 strands appear to be released from the triple helix structure than in the absence of a target for lane 7. Image J was used to grayscale analyze the S1 bands in lane 6 and lane 7, and it obtained the same results. The S1 and S1 + S2 composite strips are located very closely together, so we made auxiliary lines to help observe them. It can be seen that S1 is located below the auxiliary line (Lane 1), and the S1 + S2 complex is located above the auxiliary line (Lane 4), but the lagging band in Lane 6 and 7 seems to be just on the auxiliary line and does not perfectly coincide with S1 or S2, so we surmise that this part of the band may be mixed with S1 and S1 + S2 in different proportions. In the presence of TC, the band is roughly located in the middle of the auxiliary line, and the concentration of S1 and S1 + S2 complexes may be similar; in the absence of TC, the band is positioned slightly above the auxiliary line, and the concentration of the S1 + S2 complex may be greater than that of S1. In addition, due to the inevitable temperature increase during the glue running process, which will lead to the spontaneous opening of a small number of S1 + S2 complexes (as discussed in the simulation experiment), P1 + S2 double strands also appear in band 7.

**Figure 7:**
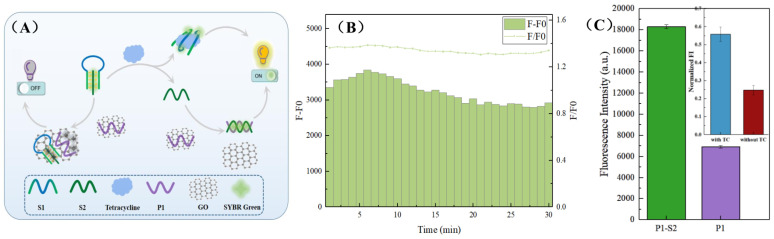
The principle (**A**) and result (**B**,**C**) of proving the feasibility with the general dye SYBR Green I.

Although GO has been widely used in biological nanodevices, some researchers have pointed out that it can adsorb ssDNA and other substances. In order to further explore the reliability and the feasibility of the fluorescence spectrum in this experiment, we changed the fluorophore on the basis of the consistency of the model to prove the feasibility from another perspective. As shown in Figure 7A, cancel the FAM fluorophore labeled by P1 and use SYBR GreenⅠ to collect the transformation between the substances. SYBR GreenⅠ is a commonly used fluorescent agent in traditional biology, and it can be embedded in the small groove of the DNA double helix structure to display thousands of times fluorescence compared with the free state. Similarly, GO can also adsorb and quench free SYBR Green I through fluorescence resonance energy transfer. Therefore, it can be distinguished whether the S2 strand is successfully released and perfectly combined with P1 according to the fluorescence changes in the system before and after adding TC. The results showed that (Figure 7B), within 30 minutes of the addition of TC, the fluorescence was significantly increased, and there was a stable fluorescence difference and ratio, indicating the formation of a stable complementary double strand. In particular, when P1 exists alone, the system exhibits extremely low fluorescence; when only the P1-S2 double strand is present, the fluorescence in the system is greatly enhanced. The fluorescence values of the two were used as the lower limit/upper limit threshold, and the fluorescence standardization map with or without TC in the complete system was plotted. It can be seen that the formation of S1–S2 structure may be weakly bound to SYBR Green I and slightly increase the fluorescence of the system; however, only in the presence of TC can the required P1-S2 double helix structure be formed and greatly enhance the fluorescence of the system.

### 3.3. Condition Optimization

By selecting appropriate reaction conditions, the intermolecular reaction can be in the best state, further reducing the mismatch rate between the biomolecules. F represents the fluorescence value in the presence of TC, F_0_ represents the initial fluorescence value in the absence of TC, while ΔF stands for the D-value between the above two elements. In the optimization experiment, the abscissa marks the different levels of each experimental factor, and the ordinate represents the fluorescence intensity (FI).

**Figure 8:**
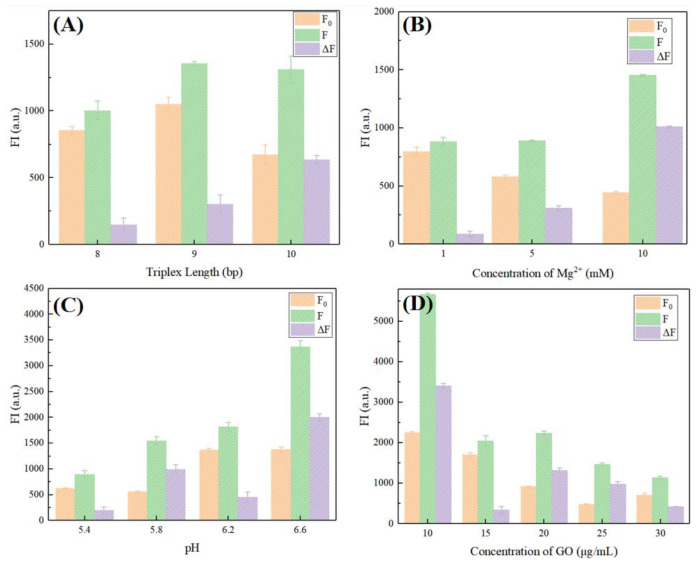
Optimization of triplex length (**A**), concentration of Mg2+ (**B**), pH value, and (**C**) concentration of GO (**D**).

Firstly, too many complementary bases in the triple helix structure may cause the triplex to be too stable and make it difficult for it to be deconstructed, so it cannot achieve the effect of rapid release of S2 strands; on the contrary, if there are too few matched bases in this part, it is not easy to form a stable triplex, which may lead to leakage and high background fluorescence. So, the length of the triple helix structure is explored by three strands named S1–8, S1–9, and S1–10. X in S1–X represents the number of base pairs that are complementarily paired between S1 and S2. According to Figure 8A, it is obvious that when the base number is 10 bp, Δ F has the highest value, so S1–10 is the best choice.

Previous studies have proved that Mg^2+^ plays an important role in the formation of the triple helix structure [32], so its concentration selection should also be carefully considered. As can be seen from Figure 8B, better fluorescence difference can be obtained only when Mg^2+^ reaches a sufficient concentration, so the Mg^2+^ concentration in the subsequent experiment is set to 10 mM.

In addition, the formation of the triple helix structure also has a lot to do with the pH value [33], so the pH value is a factor that cannot be ignored. According to the response interval of the existing Hoogsteen hydrogen bond, four different pH values were selected from 5 to 7. It was found that the background fluorescence increased in varying degrees with the increase in pH. When the pH value is 6.6, the background signal tends to be stable, while the fluorescence recovery value almost doubles, which also means that the model has better performance at this pH value. Therefore, 6.6 was selected as the optimal pH value of this model (Figure 8C).

Finally, GO plays an important role in fluorescence signal regulation in the model and directly affects the fluorescence expression level in the whole system. Therefore, its concentration needs to be optimized. It should be noted that in our previous research, fluorescence experiments were carried out with two GO dispersions (XF002-2, XF020) purchased from XFNANO with different dispersion degrees, which proved that the dispersions with a smaller and more uniform tablet diameter were more suitable for the experiment [34]. Therefore, in this paper, we continue to use the more uniform crushing GO XF020 with a sheet diameter size of <500 nm. As seen from Figure 8D, with the increase in GO concentration, the background noise is effectively reduced. Although there is a high fluorescence difference in 10 μg/mL, the accompanying high background noise makes this concentration unsuitable as the best choice. The concentration of 25 μg/mL can reduce the background noise to the lowest level, while the fluorescence recovery rate is also at a high level, so it is regarded as the optimal concentration of GO. It should be noted that when the GO concentration is higher than 25 μg/mL, F_0_ shows a slight upward trend. We surmise that this is because the GO concentration is 25 μg/mL, the adsorption of P1 has basically reached the upper limit, so a small amount of P1 is free in the solution. It has been proven that guanine can quench FAM fluorescence to some extent [35], so free P1 may be close to S1–S2 complex, and the fluorescence of labeled FAM is inhibited by guanine on the complex. When the GO concentration reaches 30 μg/mL, the probability of adsorbing the complex close to the annular part of the S1–S2 complex increases, which makes the free P1 in the system possess no additional inhibitor, so F0 increases slightly.

### 3.4. Performance Analysis

The sensitivity and specificity of the sensor are two important characteristics. Both indictors were evaluated under the optimal experimental conditions selected above. During the detection of sensitivity, TC solutions with concentrations of 0 nM, 50 nM, 100 nM, 200 nM, 300 nM, 400 nM, 600 nM, 800 nM, and 1000 nM were added respectively for fluorescence characterization under the same conditions. 

**Figure 9:**
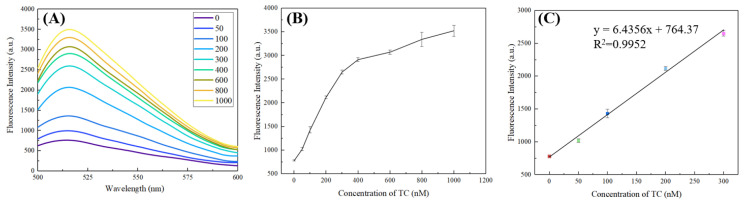
Two-dimensional simplified diagram (**A**), fluorescence value (**B**), and linear fitting results (**C**) after adding tetracycline with different concentrations.

Figure 9A shows that the fluorescence intensity generated at different concentrations can be effectively separated in the range of 0–1000 nm; simplified to Figure 9B, it can be seen that the fluorescence intensity will increase with the increase in TC solution concentration and will maintain a linear growth in the range of 0–300 nm (Figure 9C). The linear regression equation at the 518 nm emission wavelength is: y = 6.4356x + 764.37, and the determination coefficient R^2^ is 0.9952. In particular, in order to study the linear fit of the model at other emission wavelengths, we observed the R^2^ at each wavelength in the range of 500–600 nm (Figure S4). The results showed that the R^2^ at 591 nm was lower than 0.98, and most of the remaining R^2^ remained above 0.99, indicating that this method has a good linear detection capability. Using formula 3σ/S (σ is the standard deviation of the background signal and S represents the slope of the linear equation), the limit of detection (LOD) in this method is estimated to be 590 pM. Compared with other nano models used for TC detection (Table 1), the results of colorimetry can usually be directly observed with the naked eye, but it is difficult to reach a lower LOD; the electrochemical method can reach a very low LOD due to its sensitivity, but it may take a lot of time to prepare the platform in the early stage; and the fluorescence rule usually has an LOD available and a stable result output. Our design has reached a lower LOD without enzyme assistance and tedious operations, so it has certain detection advantages.

**Figure 10:**
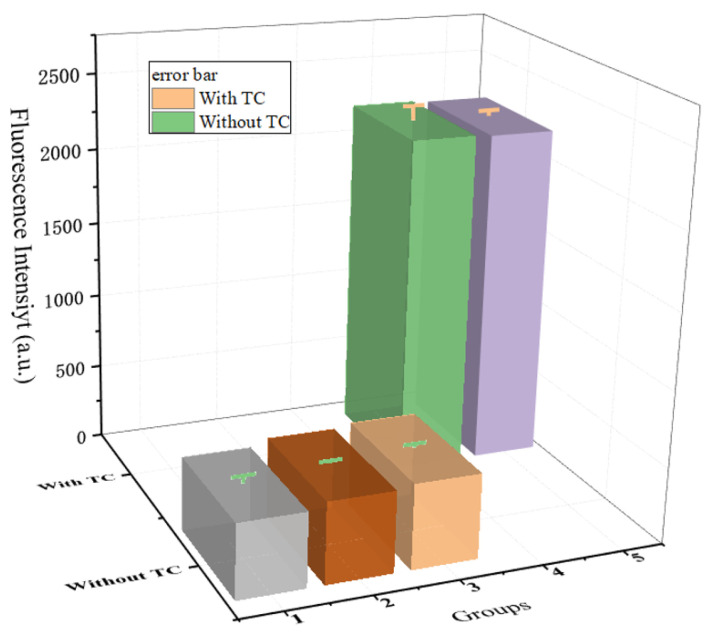
Specificity of designed components for TC detection.

It should be noted that the TC aptamer sequence used in this work was shortened from the initial 76-mer to 8-mer by Kwon et al. and had a high affinity with the four TCs [tetracycline (TC), oxytetracycline (OTC), doxycycline (DOX), and chlortetracycline (CHLOR)]. Therefore, in order to explore the selectivity of the sensor for tetracycline and the anti-interference ability of its detection, quinolone antibacterial drugs represented by ofloxacin and β-lactam antibiotics represented by amoxicillin were selected as controls. Group 1 was the blank control group, amoxicillin was added to Group 2, ofloxacin was added to Group 3, TC was added to Group 4, and group 5 was a mixture of amoxicillin, ofloxacin, and TC, so as to verify whether TC can be sensitive to detection in complex situations. The results showed that when only amoxicillin or ofloxacin was present in the component, the fluorescence intensity that the system could recover was extremely limited, which was basically the same as the background fluorescence. In addition, for TC, whether added alone or as a type of the mixture, its presence can greatly restore the fluorescence in the system. The results proved that the designed model not only has the ability to recognize TC but also has a certain anti-interference ability (Figure 10). 

In terms of universality, our team has published a study using a similar triple helix structure to detect carcinoembryonic antigen CA15-3 [39]. The difference is that the output signal used is the special fluorescence of the G-quadruplex/N-methylmesoporphyrin IX (NMM) complex. In this paper, the model was further optimized, and the introduction of GO does greatly reduce the background noise, which means that the detection of TC can reach 590 pM, and this is much lower than the EU regulation of 0.1 mg/L. However, in subsequent studies, this generic model may be modified for microdetection of other targets, so we may try to introduce signal amplification steps in the new design to further reduce detection limits and meet more detection needs. 

## 4. Conclusions

In general, we designed a nanoswitch driven by TC based on a GO–triple helix structure, and a dry experiment and wet experiment had to be used to analyze and optimize it. In detail, NUPACK and Visual DSD were used to predict the combination efficiency, composition, and stability time in the dry experiment, while the wet experiment was carried out from three aspects: feasibility, condition optimization, and performance analysis. The results show that the designed nanoswitch meets the standard LOD of 590 pM, has good selectivity, and good anti-interference ability for the detection of TC. In addition, this simple and easy-to-operate scheme has high universality; we only need to replace some sequences in S1 to detect other target substances.

## Figures and Tables

**Table 1:** Comparison of the detection methods in this article with other research methods.

Method	Simulation Experiment	LOD	Reference
Colorimetric assay	×	45.8 nM	[36]
Electrochemical assay	×	0.13 nM	[37]
Fluorescent assay	×	0.97 nM	[3]
Fluorescent assay	×	6.49 nM	[38]
Fluorescent assay	√	0.59 nM	This work

## Supplementary Materials

The following are available online at https://www.mdpi.com/article/10.3390/mi13122119/s1, Figure S1: NUPACK results of S1-rTC (A), and cTC-rTC (B), Figure S2: NUPACK result at actual concentration, Figure S3: Visual DSD result at actual concentration, Figure S4: 3D spectral scanning diagram and correlation coefficient at each emission wavelength, and Table S1: DNA sequences.
