# Supplementary material for: Potential Universal Engineering Component: Tetracycline Response Nanoswitch Based on Triple Helix-Graphene Oxide
Source: Micromachines (Basel). 2022 Nov 30;13(12):2119. doi: 10.3390/mi13122119 (PMC9784820; doi:10.3390/mi13122119)
Supplement: Supplementary file 1 [file micromachines-13-02119-s001.zip › micromachines-1968018-supplementary.pdf]

Supporting Information

Table S1. DNA sequences

Type of strand	Strand sequences (5’ → 3’)
S1-8	TCTCTCCTCGGTGGTGTCCTCTCT
S1-9	CTCTCCTTTCGGTGGTGTTTCCTCTC
S1(S1-10)	TCTCTCCTTTCGGTGGTGTTTCCTCTCT
S2	AGAGAGAGGAAA
P1	FAM-TTTCCTCTCTCT

In NUPACK, only strand to strand combination can be simulated. In order to verify whether the complementary sequence of TC aptamer (the aptamer named cTC, the complementary sequence named rTC) can replace the role of TC, calculation and analysis are carried out: Use NUPACK to simulate the combination of S1-rTC and cTC-rTC respectively (Fig. S1), their free energy is obtained from Formula $\Delta G(s) = \sum_{loop \in s} \Delta G(\text{loop})$. From $\triangle G = -RTlnK$, we can get their dissociation constant Kd are 6.09×10^{-10}M and 7.82×10^{-10}M (0.609 nM and 0.782 nM). Kown et al. [29] proves that the Kd of the used aptamer cTC and TC is 1.067 nM, comparing Kd in several cases, it can be seen that the binding efficiency of S1-rTC, cTC-rTC and cTC-TC is S1-rTC>cTC-rTC>cTC-TC from large to small, and the Kd values of the three are roughly in the same order of magnitude. In combination with another adtamer alternative (Kd=3.413nM) mentioned by Kown et al.[29], which is used together with cTC for TC colorimetric identification, the results are roughly consistent. Therefore, we believe that the complementary sequence of the aptamer can be used to replace the target.

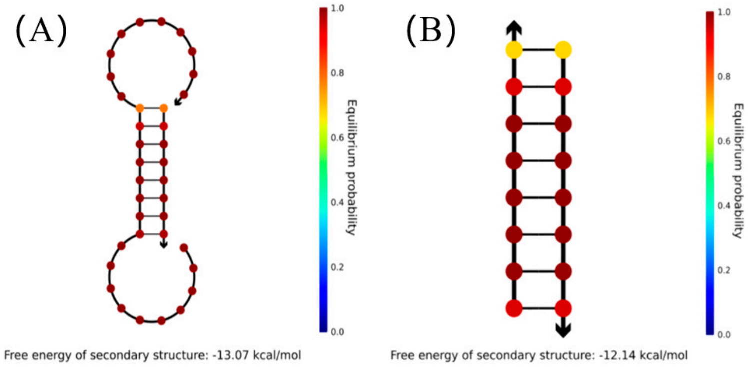

Figure S1. Nupack results of S1-rTC (A), and cTC-rTC (B).

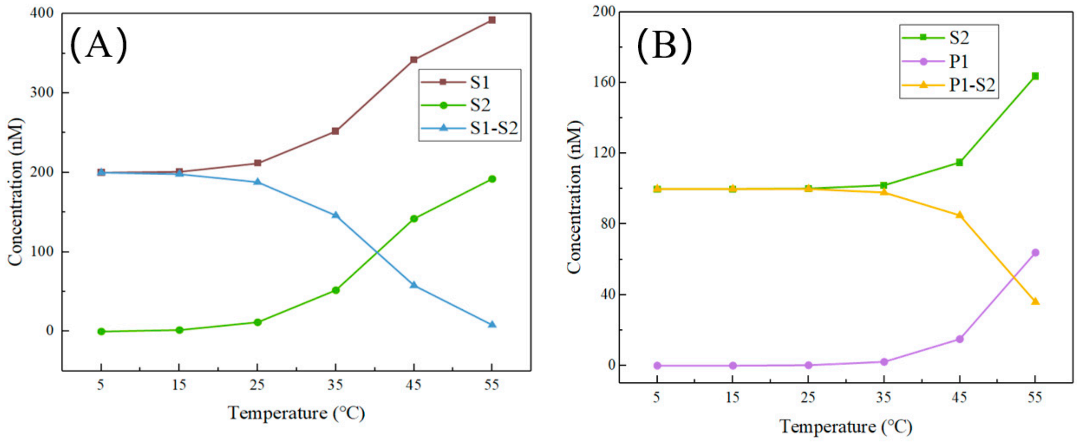

Figure S2. NUPACK result at actual concentration

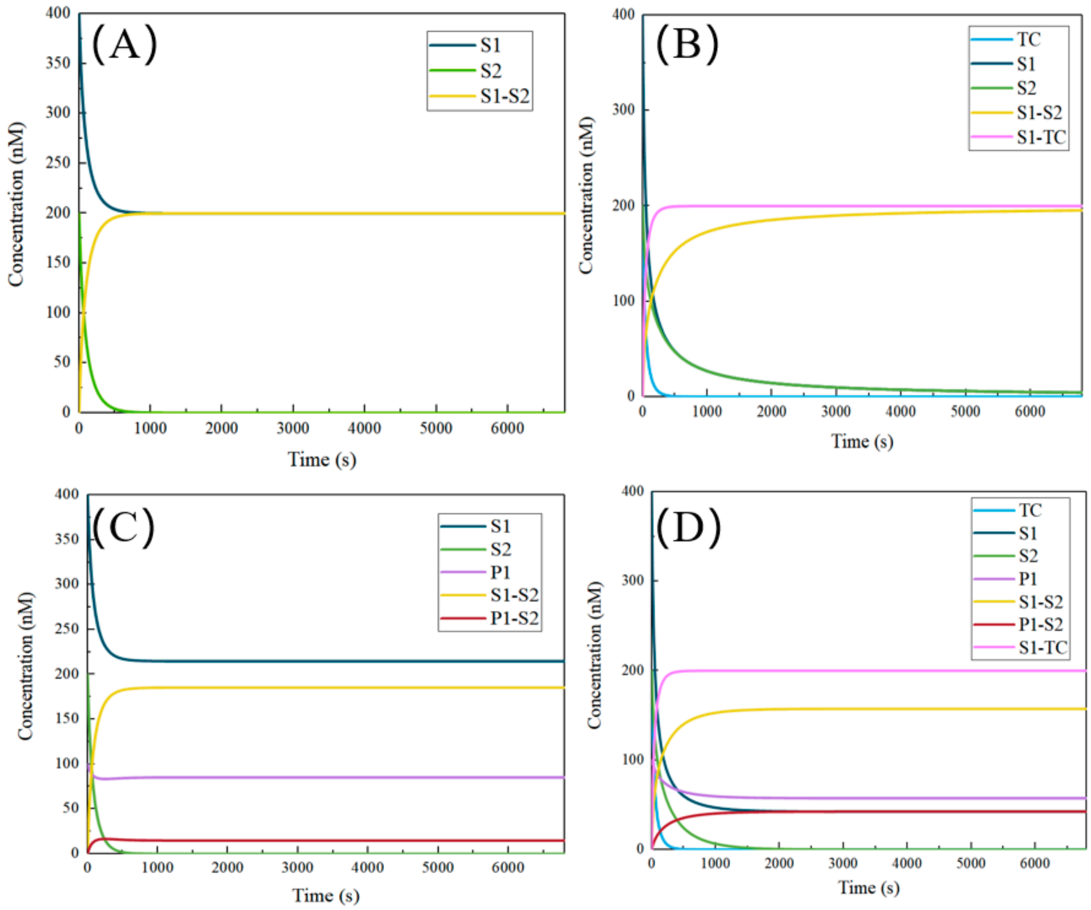

Figure S3. Visual DSD result at actual concentration

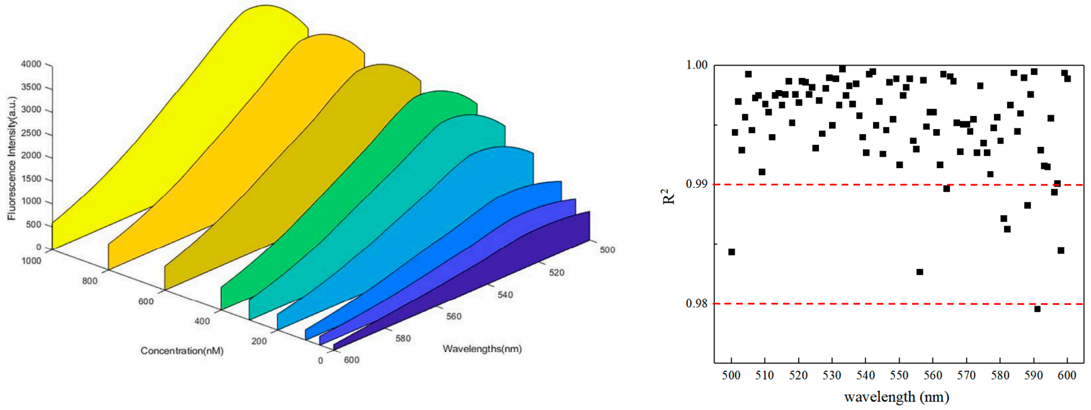

Figure S4. 3D spectral scanning diagram and correlation coefficient at each emission wavelength
